# Fine root morphological characteristics and biomass distribution characteristics of different artificial forests in coastal salt land and their influencing factors

**DOI:** 10.3389/fpls.2025.1633856

**Published:** 2025-09-24

**Authors:** Zhibao Wang, Jing Liang, Hong Jiang, Xiangbin Gao, Shouchao Yu, Chuanjie Zhou, Yuwei Guo

**Affiliations:** ^1^ Agricultural Science and Engineering School, Liaocheng University, Liaocheng, China; ^2^ Soil Research Institute, Shanghai Academy of Landscape Architecture Science and Planning, Shanghai, China; ^3^ College of Pharmacy, Guizhou University of Traditional Chinese Medicine, Guiyang, China

**Keywords:** tree species, fine root traits, soil properties, ecological adaptation, coastal saline soil

## Abstract

To elucidate the ecological adaptations of fine root morphological traits and biomass in tree species with different life forms to coastal saline soil, five species (*Robinia pseudoacacia*(RP), *Sapium sebiferum*(SA), *Salix matsudana*(SM), *Quercus virginiana*(QV), *Ligustrum lucidum*(LI)) were investigated using continuous root coring. Fine root morphological traits, biomass distribution, and their relationships with soil water, temperature, electrical conductivity, pH, total nitrogen, total phosphorus, and soil organic matter were analyzed to reveal species-specific adaptation strategies. Results showed significant differences (*P*<0.05) in fine root morphological indices (specific root length, specific root surface area, root length density, and root surface area density). *RP* exhibited the highest specific root length (135.38 m·g^-1^) and specific root surface area (1141.07 cm²·g^-1^), while *QV* showed the lowest values (39.17 m·g^-1^ and 315.22 cm²·g^-1^, respectively). Both root length density and root surface area density decreased with increasing soil depth. Fine root biomass differed significantly among species (*P*<0.01), with *LI* having the highest biomass (273.42 g·m^-2^) and *RP* the lowest (77.05 g·m^-2^). Vertically, biomass declined with depth; horizontally, it decreased with distance from the trunk. Root extinction coefficients indicated *QV* and *RP* as deep-rooted species, while *LI*, *SM* and *SA* were shallow-rooted. Seasonal dynamics revealed unimodal patterns in live and dead fine root biomass for *RP*, *LI*, *QV*, and *SA*. In contrast, *SM* exhibited a unimodal pattern in live fine root biomass but a distinct bimodal pattern in dead fine root biomass. Correlation analysis identified soil electrical conductivity, soil water, and total nitrogen as primary environmental drivers of fine root traits and biomass.

## 1 Introduction

Currently, global soil salinization is increasingly severe, posing a worldwide ecological and socioeconomic challenge (Chi et al., 2012; Bui, 2013; Wang et al., 2022). There are approximately 950 million hectares of saline-alkali land worldwide, with about 35 million hectares in China (Zhang, 2008; Wang, 2020). A portion of these saline lands is formed by dredging seabed sediments and filling them onto land using dredgers (e.g., the coastal new areas of Tianjin, Guangzhou, and Shanghai). In addition to the characteristics of high soil salinity and pH typical of general saline-alkali soils, such saline soils also feature high bulk density and poor nutrient content. Furthermore, due to the low groundwater level of coastal saline soils and the influence of seawater tides on coastal soils, the moisture and salinity of coastal saline soils fluctuate periodically, resulting in extremely complex soil properties, which severely affect the normal growth of landscaping plants (Wu et al., 2009; He et al., 2014). Therefore, studying plant adaptation mechanisms to saline-alkali soils holds significant theoretical and practical importance for screening and cultivating superior greening species.

Fine roots (φ ≤2 mm) are the most active and sensitive organs in plant root systems and are highly susceptible to soil environmental influences (Steele et al., 1997; Huang et al., 2008; Han et al., 2018). The spatiotemporal dynamics and distribution patterns of fine root morphological traits and biomass are critical quantitative indicators for assessing fine root responses to environmental stress (Violle et al., 2007; Bardgett et al., 2014). These traits are influenced by abiotic factors such as soil moisture, temperature, salinity, and nutrient availability, as well as interspecific differences (Xu et al., 2020). In soil environments, root systems of different tree species exhibit distinct characteristics (deep-rooted *vs*. shallow-rooted) and distribution patterns (vertical *vs*. horizontal). Due to differences in the strategies (acquisitive *vs*. conservative) adopted by fine roots of plants for soil resource utilization, changes in soil environment often lead to alterations in the distribution patterns of fine roots. For example, in arid stress environments, roots absorb water from deep soil by expanding their growth depth in the soil, and increase their own fine root biomass and root length to survive, reflecting the explorative nature of roots (Bergmann et al., 2020; Lin et al., 2020). A high-salinity soil environment can cause water loss in plants and restrict the root function of absorbing water and nutrients, leading to changes in the growth depth and morphological characteristics of fine roots (Ashraf and Harris, 2004; Grady et al., 2005; Flowers and Colmer, 2008; Turkey, 2012; Imada et al., 2013). In addition, a high-salinity soil environment can cause the differentiation of root spatial niches (Jiang et al., 2016). Meanwhile, plants adapt to salt stress environments by reducing total fine root biomass and increasing dead fine root biomass, reflecting the conservative nature of roots (Hasegawa et al., 2000). All the above root adaptation strategies follow the root economics spectrum theory (Bergmann et al., 2020). Since soil salinity exhibits periodic changes under the influence of solar radiation and precipitation (He et al., 2015), it can also affect the periodic changes of fine root biomass (Hendrick and Pregitzer, 1993; Burke and Raynal, 1994; Quan et al., 2010). To adapt to changes in the soil salinity environment, plant roots will select survival strategies with the minimum cost and maximum benefit based on their plastic characteristics and the “cost-benefit” theory (Eissenstat, 1997; Bardgett et al., 2014; Ding et al., 2022; Li et al., 2022; Sun et al., 2022), specifically by adjusting the allocation pattern of fine root biomass and changing fine root morphological characteristics to adapt to the environment (Li et al., 2017; Burton et al., 2000; Li et al., 2022; Wen et al., 2022). Therefore, studying the relationship between fine root functional traits and soil environmental factors is helpful for revealing the ecological adaptability of fine roots to saline environments.

Most studies on plant root-soil environmental adaptations have focused on natural ecosystems (Fitter and Stickland, 1991), with limited research on artificial plantations in coastal saline soils under humid climates in China. Coastal saline soils are characterized by high salinity, elevated pH, and low nutrient availability. In such unique environments, questions remain: How do fine root morphological traits and spatiotemporal biomass distribution vary among tree species? What are the relationships between fine root biomass, morphological traits, and soil environments? Therefore, studying the relationships between fine root characteristics of different tree species and soil physicochemical properties can reveal species-specific adaptation strategies to saline soils, providing critical guidance for afforestation in saline-alkali areas.

Accordingly, based on the different life forms of tree species, five tree species, including evergreen species *Quercus virginiana* (QV)and *Ligustrum lucidum* (LI), as well as deciduous species *Robinia pseudoacacia*(RP), *Sapium sebiferum*(SA), and *Salix matsudana*(SM), were selected as research objects. The spatiotemporal variations in fine root biomass, the distribution patterns of morphological characteristics, and their influencing factors among different tree species were analyzed, aiming to reveal the ecological adaptation mechanisms of different tree species to saline-alkali soil environments. The research results will help deepen the understanding of root biology and root physiological ecology theories, and provide theoretical basis and technical support for formulating greening strategies in saline-alkali land environments.

## 2 Materials and methods

### 2.1 Study area

**Figure 1:**
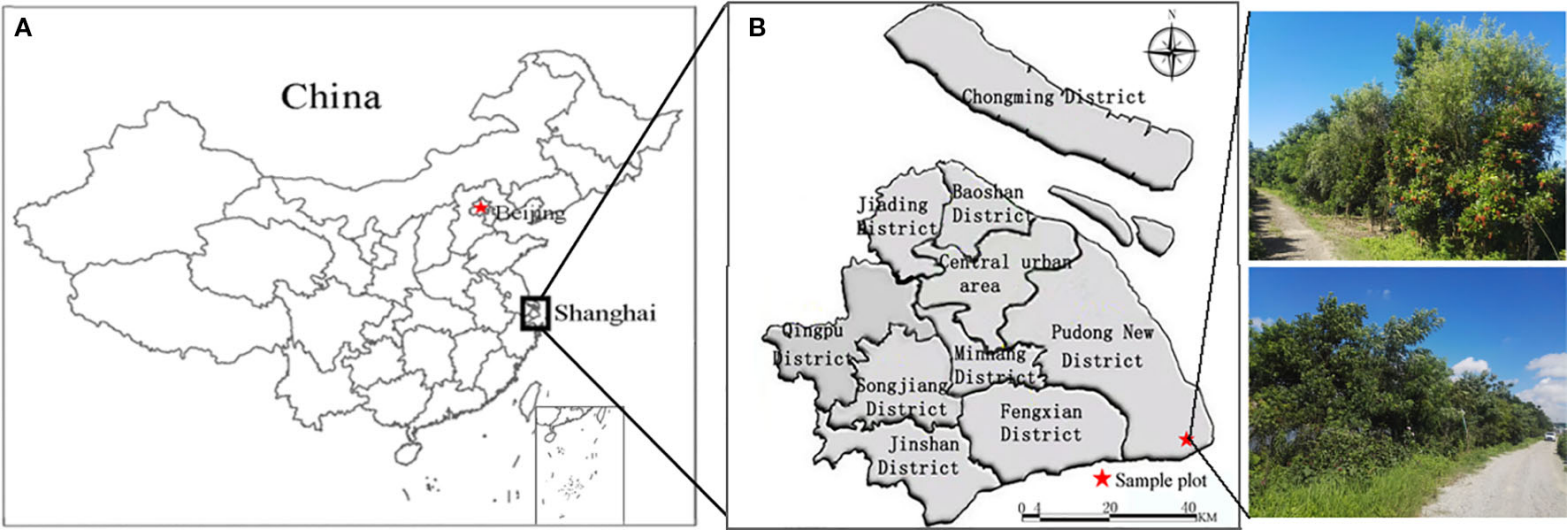
Study area. **(a)** Shanghai, **(b)** research area in Shanghai.

The experimental site is located in Lingang New City, Shanghai (120°53′–121°17′ E, 30°59′–31°16′ N), characterized by a subtropical maritime climate with warm and humid conditions, abundant rainfall, ample sunshine, and distinct seasons. The mean annual temperature ranges from 15.2 °C to 15.8 °C, with the coldest month (January) averaging 3.1–3.9 °C. Annual precipitation is 900–1050 mm, with 60% concentrated between May and September. Monthly evaporation averages 91.9 mm, annual sunshine duration is 2000–2200 h, and mean relative humidity is 77–83%. The soil is predominantly saline-alkali, with high salinity (≥0.4%, dominated by Na^+^ and Cl^−^), high groundwater table, elevated pH (pH >8.5 in 97.5% of the area), low organic matter content (<20 g·kg^-1^), poor aeration, shallow groundwater depth (0.5–2.5 m), and high groundwater mineralization, creating harsh conditions for plant growth. The experimental area spans 900 m in length, 10 m in average width, and 9000 m² in total area (**Figure 1**).

### 2.2 Experimental design and sampling

Species Selection: In January 2018, two evergreen species (QV, LI) and three deciduous species (RP, SA, SM) were selected based on life forms. The five species had comparable mean tree height, diameter at breast height, and crown width (**Table 1**). Three trees per species were sampled as experimental replicates, spaced ≥15 m apart to avoid root interference. Herbaceous plants were manually removed throughout the experiment to eliminate their impact on root growth and sampling.

**Table 1:** Basic characteristics of different tree species.

Tree species	Year (a)	Height (m)	DBH (cm)	Crown width (m)
RP	8	5.45 ± 0.25	10.38 ± 0.21	3.10 ± 0.30
SA	8	5.50 ± 0.30	11.11 ± 0.10	3.10 ± 0.50
SM	8	5.80 ± 0.50	11.43 ± 0.17	3.10 ± 0.20
QV	8	5.10 ± 0.45	10.56 ± 0.15	3.30 ± 0.30
LI	8	5.37 ± 0.31	10.01 ± 0.27	3.31 ± 0.25

RP, Robinia pseudoacacia; SA, Sapium sebiferum; SM, Salix matsudana; QV, Quercus virginiana; LI, Ligustrum lucidum.

**Figure 2:**
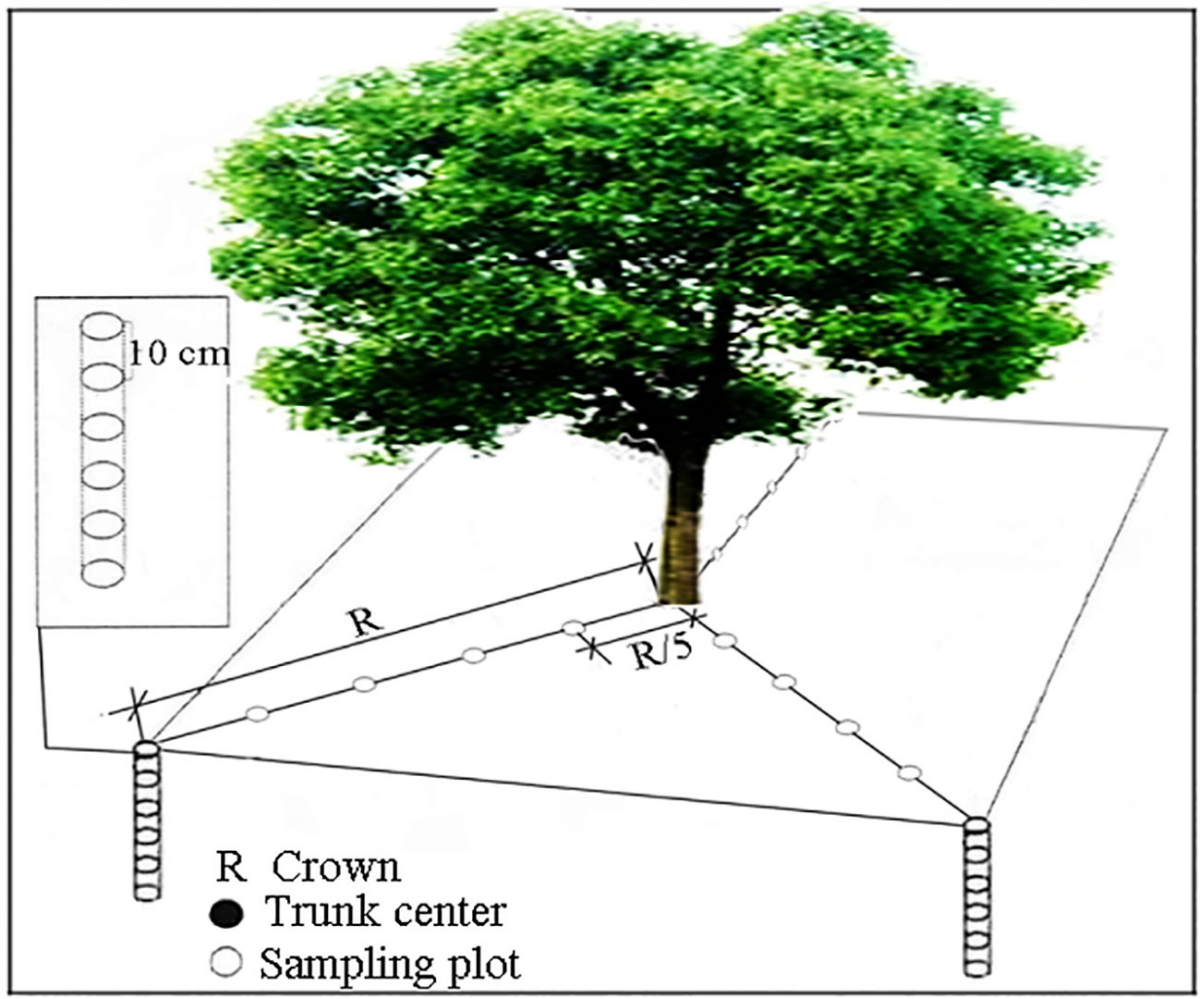
Fine-root sampling diagram.

Sampling Method: For each species, soil cores were collected using a root auger (internal diameter φ =5.0 cm, height H = 10 cm) at five equidistant points (30 cm intervals) along three radial directions from the trunk center to the canopy edge, with a sampling boundary at 1.5 m from the trunk. Soil samples were taken at 50 cm depth, divided into five layers (0–10 cm, 10–20 cm, 20–30 cm, 30–40 cm, 40–50 cm) (**Figure 2**; Jian et al., 2015). Soil electrical conductivity (EC) and temperature were measured *in situ* for each layer using a calibrated electrical conductivity Tester 11+ (Spectrum Technologies Inc., USA). Three soil cores from the same layer and distance were pooled to extract roots, and a composite soil sample was collected. Roots and soil samples were labeled, stored in sealed bags, and transported to the laboratory for analysis. The remaining soil was backfilled into the original holes. The collection of root and soil samples was completed between the 5th and 7th days of each even month under rainless and snowless weather conditions, with the sampling period lasting from February 2018 to February 2019.

### 2.3 Fine root processing

Roots were rinsed with tap water using a 40-mesh sieve (aperture 0.42 mm) until free of soil. Clean roots were blotted dry with absorbent paper, placed in plastic bags, and stored at 4°C. Fine roots (φ ≤2 mm) were isolated using forceps and calipers (Wang et al., 2016), with non-target roots (e.g., grass roots) discarded. Live and dead fine roots were differentiated based on morphology, color, odor, and elasticity (Noguchi et al., 2007; Imada et al., 2015; Ma and Chen, 2017).

### 2.4 Fine root parameter calculations

Processed roots were scanned using a Win-RHIZO 2005C root analyzer (Regent Instruments Inc., Quebec, Canada) to obtain mean diameter, surface area, volume, and length. After scanning, roots were oven-dried at 80 °C to constant weight for calculating the following parameters: fine root biomass (FRB), root tissue density (RTD), root length density (RLD), specific root length(SRL), specific root surface area (SRSA), and root surface-area density (RSAD) (Brédoire et al., 2016) in each soil layer, as follows:


(1) $$\text{FRB }\left(\text{g}\cdot {\text{m}}^{-2}\right)=M\left(\text{g}\right)\times 10^{4}/[\pi {\left(\text{d}/2\right)}^{2}]$$

(2) $$\text{RTD }\left(\text{g}\cdot {\text{cm}}^{-3}\right)=M\left(\text{g}\right)/\text{fine root volume }\left({\text{cm}}^{3}\right)$$

(3) $$\text{RLD }\left(\text{m}\cdot {\text{m}}^{-3}\right)=L\left(\text{m}\right)\times 10^{6}/[\pi {\left(\text{d}/2\right)}^{2}\times \text{h}]$$

(4) $$\text{SRL }\left(\text{m}\cdot {\text{g}}^{-1}\right)=L\left(\text{m}\right)/\text{fine root mass }\left(\text{g}\right)$$

(5) $$\text{SRSA }\left({\text{cm}}^{2}\cdot {\text{g}}^{-1}\right)=S\left({\text{cm}}^{2}\right)/\text{fine root mass }\left(\text{g}\right)$$

(6) $$\text{RSAD }\left({\text{m}}^{2}\cdot {\text{m}}^{-3}\right)=S\left({\text{m}}^{2}\right)\times 10^{6}/[\pi {\left(\text{d}/2\right)}^{2}\times \text{h}]$$

w*here d* is auger internal diameter (cm), and *h* is auger height (cm). *M* = fine root dry weight; *L* = total length of fine root; *S* = the length of dry fine root surface area).

### 2.5 Root extinction coefficient (*β*)

The vertical root distribution model proposed by Gale and Grigal (1987) was applied:


(7) $$Y=1–\beta^{d}$$

*where Y* is cumulative percentage of root biomass from the surface to depth *d* (cm). The *β* value mainly represents the relationship between the vertical distribution of the root system and the depth of the soil layer. A higher *β* value indicates greater root biomass in deeper soil layers. A smaller *β* value indicates that the plant root system is closer to the surface soil layer.

### 2.6 Soil property analysis

After gravel, tree roots, and grass roots were removed from the collected soil samples, the soil water content was determined using the oven-drying method. Soil samples from each layer were placed into three aluminum boxes, and the wet weight (m_1_) was measured using an electronic balance (accuracy: 0.001 g). The boxes containing moist soil were then oven-dried at 105 °C until constant weight was achieved. After cooling, the dry weight (m_2_) was recorded, and the empty box weight (m_0_) was subtracted. Soil water content (W) was calculated using the following formula:


(8) $$W=\frac{m_{1}-m_{2}}{m_{2}-m_{0}}\times 100\%$$

Soil electrical conductivity and temperature were measured using a portable conductivity meter (electrical conductivity Tester 11+, Spectrum Technologies Inc., USA). For each soil layer, measurements were taken at three positions (upper, middle, and lower) within the soil core. The average of the three electrical conductivity and temperature readings was recorded as the representative value for the respective layer.

Soil pH was determined by the potentiometric method. Air-dried soil was ground and passed through a 100-mesh sieve before being placed in sealed bags. In the laboratory, 20 g of soil sample was weighed using an electronic balance (accuracy: 0.001 g), then mixed thoroughly with 50 ml of deionized water. The mixture was shaken using a mechanical shaker at 225 rpm for 5 minutes. After shaking, the suspension was allowed to settle for 30 minutes, and soil pH was measured using a calibrated soil pH meter (pH7110, WTW, Germany). Three replicates were prepared for each soil layer, with each replicate measured three times. The average of the three measurements was recorded as the soil pH for that layer. Soil organic matter content was determined by the potassium dichromate external heating method. Soil total nitrogen content was determined by the Kjeldahl method. Soil total phosphorus content was determined by the molybdenum-antimony anti-colorimetric method.

### 2.7 Data processing

Data organization and statistical analysis were performed using Microsoft Excel software to compile fine root parameters (fine root biomass, specific root length, root length density, etc.) and soil physicochemical properties (soil water, temperature, electrical conductivity, pH, total nitrogen, total phosphorus, etc.), with calculation of means and standard deviations.

Statistical analyses were conducted using Statistical Product and Service Solutions (SPSS) 16.0 software. Prior to analysis, homogeneity of variance was tested for all data; logarithmic transformation was applied when variances were unequal. One-way analysis of variance (One-way ANOVA) and the least significant difference (LSD) method were employed to examine differences in fine root biomass and morphological indices among different tree species. Multifactor analysis of variance was used to analyze the effects of tree species, soil depth, and their interaction on fine root biomass and morphological characteristics. The significance level was set at α ≤ 0.05. All analytical figures were generated using Origin Pro 9.0 software.

## 3 Results

### 3.1 Fine root morphological characteristics of different tree species

Significant differences (*P*< 0.05) were observed among tree species in fine root average length, surface area, volume, diameter, root length density, specific root length, specific root surface area, and root surface area density. Across soil layers, significant differences (*P*< 0.05) were found in average length, surface, volume, root length density, and root surface area density, while no significant differences (*P* > 0.05) were detected in average diameter, specific root length, and specific root surface area. Except for root tissue density, the interaction between tree species and soil depth had no significant effect on the aforementioned fine root characteristics(*P* > 0.05, **Table 2**).

**Table 2:** Results of ANOVA of the effects of tree species and soil depths on the morphological characteristics of fine roots.

Parameter	Source of variation					
	Tree species		Soil depth		Tree species×Soil depth	
	*F*	*P*	*F*	*P*	*F*	*P*
Length (cm)	7.75	<0.01**	3.03	<0.05*	0.86	Ns
Surface area (cm^2^)	5.55	<0.01**	2.86	<0.05*	0.83	Ns
Volume (cm^3^)	4.48	<0.05*	2.53	<0.05*	0.73	Ns
Diameter (mm)	8.23	<0.01**	2.36	Ns	1.51	Ns
Root tissue density (g·cm^-3^)	70.20	<0.01**	2.34	Ns	2.21	<0.05*
Root length density (m·m^-3^)	7.75	<0.01**	3.03	<0.05*	0.68	Ns
Specific root length (m·g^-1^)	3.54	<0.05*	1.21	Ns	0.73	Ns
Specific root surface area (cm^2^·g^-1^)	2.76	<0.05*	1.24	Ns	0.69	Ns
Root surface area density (cm^2^·m^-3^)	5.56	<0.01**	2.87	<0.05*	0.83	Ns

**At 0.01 level (double side) significant difference, *at 0.05 level (double side) significant difference. NS, there was no significant difference.

**Figure 3:**
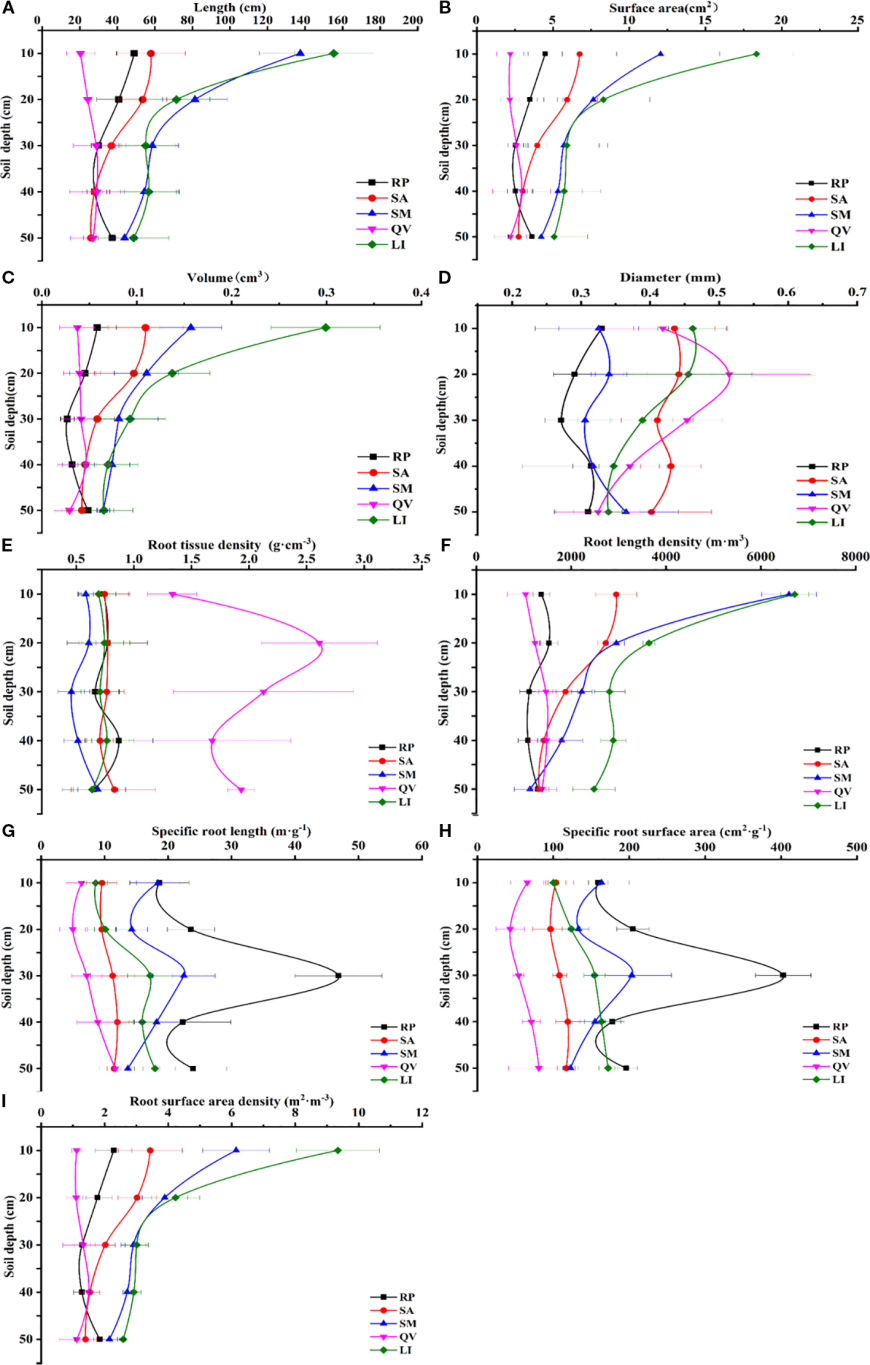
The changes of fine root morphological characteristics of different tree species in different soil depths. **(a)** fine root length, **(b)** fine root surface area, **(c)** fine root volume, **(d)** fine root diameter, **(e)** fine root tissue density, **(f)** fine root length density, **(g)** specific root length, **(h)** specific root surface area, **(i)** root surface area density. RP, *Robinia pseudoacacia*; SA, *Sapium sebiferum*; SM, *Salix matsudana*; QV, *Quercus virginiana*; LI, *Ligustrum lucidum.*

Root length density and root surface area density are the most important morphological indicators of fine roots. Higher root length density and specific surface area indicate stronger capacity for water and nutrient absorption. The study found that the five tree species showed different vertical distribution patterns of root length density and root surface area density (**Figure 3**). For root length density: LI, SA and SM showed maximum root length density and root surface area density in surface soil (0–10 cm), which decreased with increasing soil depth. RP had maximum root length density (1533.80 m·m^-3^) in 10–20 cm layer. QV showed an increasing root length density from 0–10 cm to 30–40 cm, peaking at 1486.74 m·m^-3^ in 30–40 cm layer, then decreasing. For root surface area density: RP showed maximum root surface area density (2.287 m·m^-3^) in 0–10 cm layer, decreasing to 30–40 cm then increasing in 40–50 cm. SA and SM showed decreasing root surface area density with depth, with maxima (3.43 m·m^-3^ and 6.13 m·m^-3^ respectively) in 0–10 cm layer. QV showed an increasing root surface area density from 0–10 cm to 30–40 cm, then decreasing to 40–50 cm.

The five species showed consistent overall trends in specific root length and specific root surface area (**Figure 3**). In total values: RP had highest specific root length (135.38 m·g^-1^) and specific root surface area (1141.07 cm^2^·g^-1^). QV showed lowest specific root length (39.17 m·g^-1^) and specific root surface area (315.22 cm^2^·g^-1^). Vertically: LI, SA and QV showed increasing specific root length and specific root surface area from 0–10 cm to 40–50 cm, peaking at 40–50 cm with specific root length of 17.94 m·g^-1^, 11.49 m·g^-1^ and 11.69 m·g^-1^ respectively, and specific root surface area of 172.26 cm^2^·g^-1^, 117.14 cm^2^·g^-1^ and 81.04 cm^2^·g^-1^ respectively. RP and SM showed maximum specific root length (46.87 m·g^-1^ and 22.50 m·g^-1^ respectively) and specific root surface area (402.91 cm^2^·g^-1^ and 203.42 cm^2^·g^-1^ respectively) in 20–30 cm layer.

### 3.2 Spatiotemporal distribution of fine root biomass

#### 3.2.1 Vertical distribution of fine root biomass

Significant differences (*P*<0.01) were observed among tree species in live fine root biomass, dead fine root biomass and total fine root biomass (**Table 3**). Significant differences were also found across soil layers for live fine root biomass and total fine root biomass (*P*<0.01), while dead biomass varied significantly (*P*<0.05). The interaction between tree species and soil depth significantly affected live fine root biomass and total fine root biomass (*P*<0.01) and dead fine root biomass (*P*<0.05) (**Table 3**).

**Table 3:** Results of ANOVA of the effects of tree species and soil depths on fine root biomass.

Parameter	Source of variation		
	Tree species	Soil depth	Tree species×Soil depth
Live fine root biomass (g·m^-2^)	13.56**	13.09**	4.09**
Dead fine root biomass (g·m^-2^)	59.84**	4.72*	2.25*
Total fine root biomass (g·m^-2^)	19.73**	15.02**	4.19**

**At 0.01level (double side) significant difference, *at 0.05 level (double side) significant difference.

**Figure 4:**
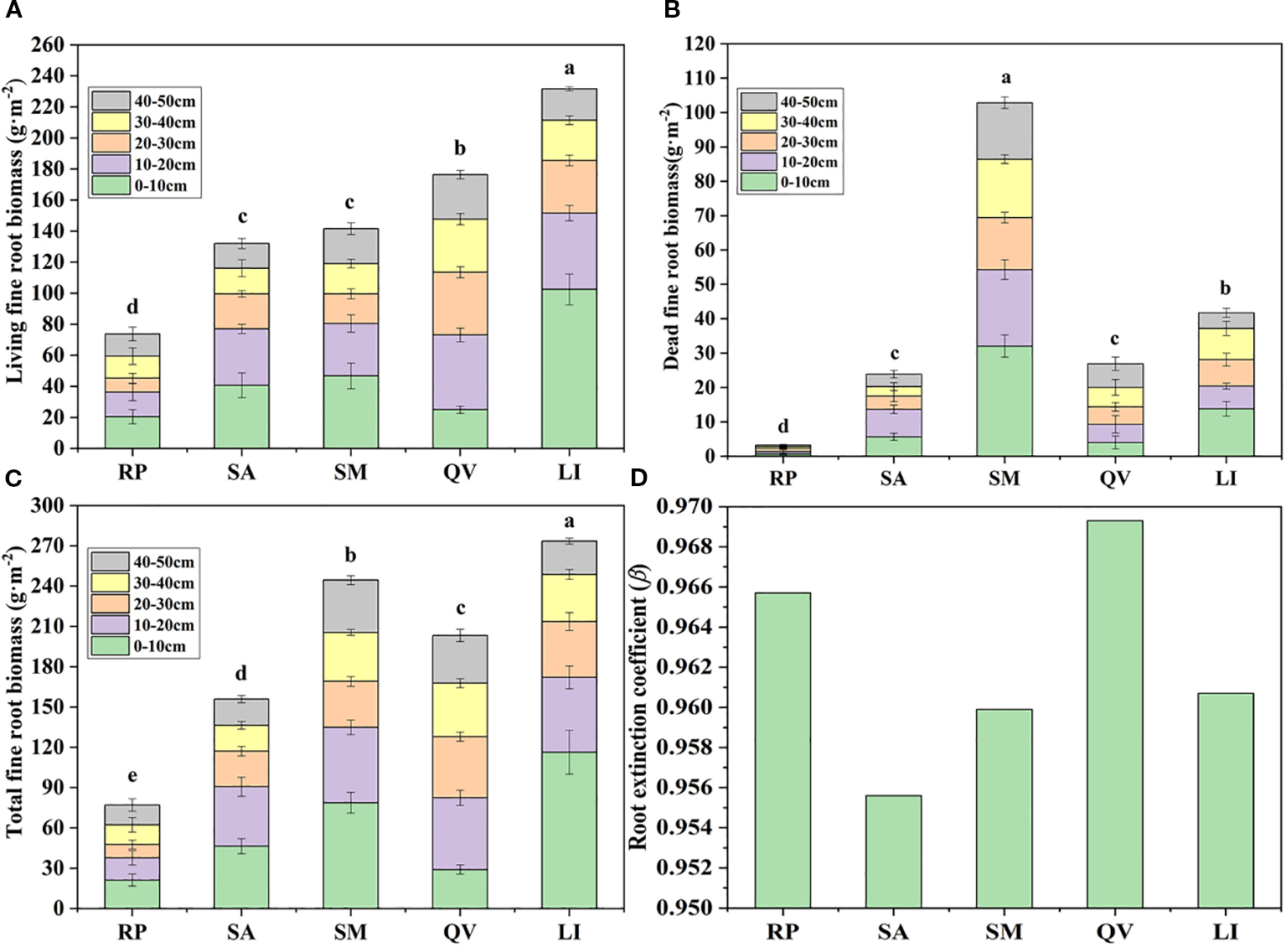
Comparison of the fine root biomass in different tree species. **(a)** living fine root biomass, **(b)** dead fine root biomass, **(c)** total fine root biomass, **(d)** root extinction coefficient. RP, *Robinia pseudoacacia*; SA, *Sapium sebiferum*; SM, *Salix matsudana*; QV, *Quercus virginiana*; LI, *Ligustrum lucidum.* There was significant difference in cumulative biomass of different tree species with different letters, but there was no significant difference with the same letter (*P*< 0.05).

The five tree species exhibited significant differences in live fine root biomass (*P*< 0.05), with LI showing the highest value (231.68 g·m^-2^) and RP the lowest (73.78 g·m^-2^) (**Figure 4a**). Similarly, dead fine root biomass varied significantly (*P*< 0.05), peaking in SM (102.87 g·m^-2^) and reaching its minimum in RP (3.27 g·m^-2^) (**Figure 4b**). Total fine root biomass also differed significantly (*P*< 0.05), being highest in LI (273.42 g·m^-2^) and lowest in RP (77.05 g·m^-2^) (**Figure 4c**). In the 0–10 cm soil layer, RP, SA, SM, and LI accounted for 27.51%, 29.78%, 32.23%, and 42.56% of their total biomass respectively, while QV showed maximum biomass (53.44 g·m^-2^, 26.29% of total) in the 10–20 cm layer. Overall, 61.86% (RP), 75.15% (SA), 69.20% (SM), 62.93% (QV), and 78.15% (LI) of total fine root biomass was concentrated in the upper 0–30 cm soil layer.

**Figure 5:**
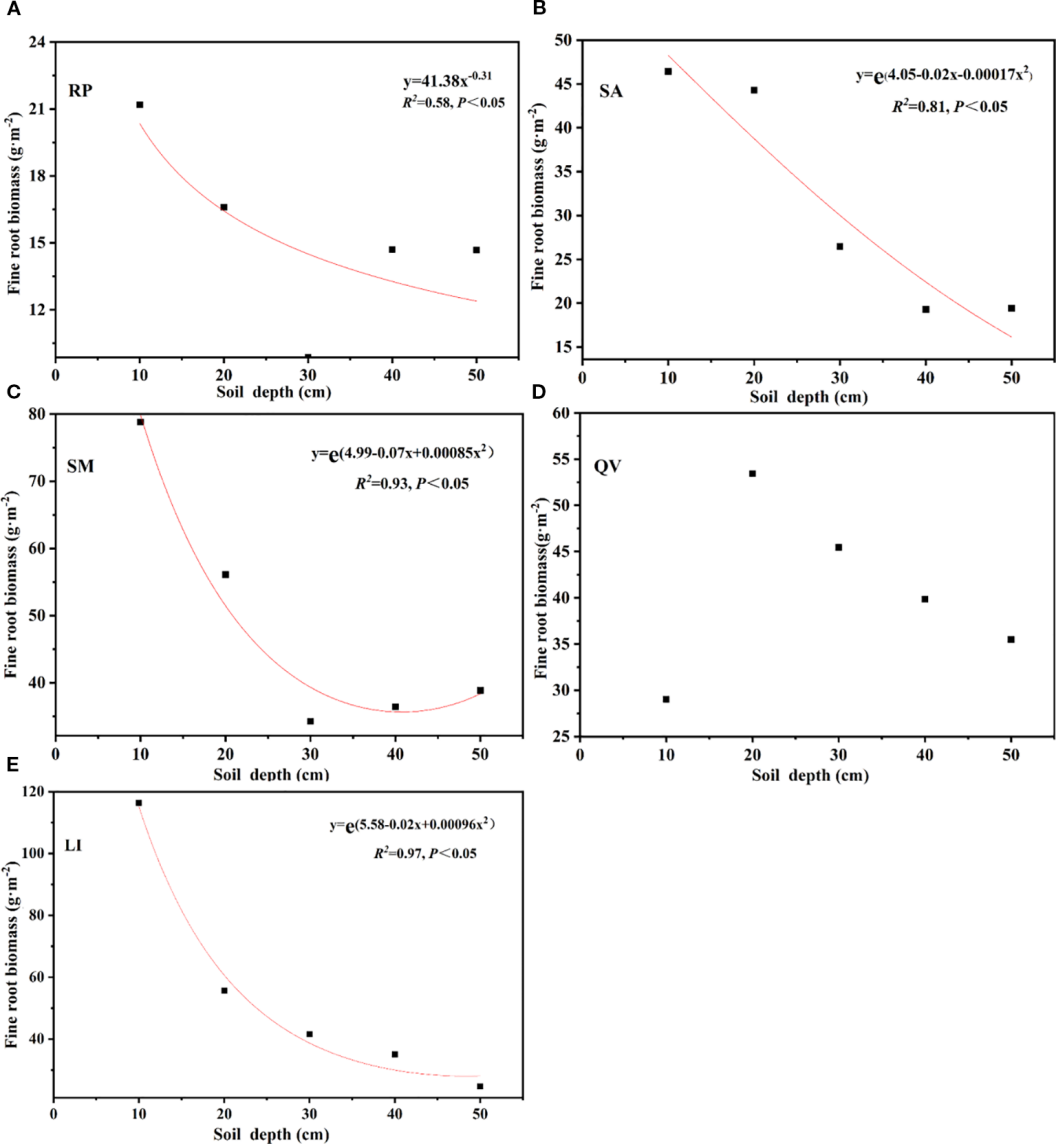
Fitting function of fine root biomass of different tree species with the soil depth. **(a)** RP, **(b)** SA, **(c)** SM, **(d)** QV, **(e)** LI. RP, *Robinia pseudoacacia*; SA, *Sapium sebiferum*; SM, *Salix matsudana*; QV, *Quercus virginiana*; LI, *Ligustrum lucidum.*

The β of the five tree species revealed distinct rooting patterns: QV (0.9693) and RP (0.9657) exhibited higher values, indicating their classification as deep-rooted species, while LI (0.9607), SM (0.9599), and SA (0.9556) showed progressively lower coefficients, confirming their status as shallow-rooted species (**Table 4**, **Figure 4d**). Linear regression analysis demonstrated that followed an exponential decay pattern with increasing soil depth for all species except QV (*P*<0.05, **Figure 5**), further supporting the differential vertical distribution strategies among these tree species in coastal saline soils.

**Table 4:** Root extinction coefficient of fine root total biomass in different tree species.

Tree species	Root extinction coefficient(*β*)
RP	0.9657
SS	0.9556
SM	0.9599
QV	0.9693
LI	0.9607

RP, Robinia pseudoacacia; SA, Sapium sebiferum; SM, Salix matsudana; QV, Quercus virginiana; LI, Ligustrum lucidum.

#### 3.2.2 The horizontal distribution of fine root biomass of different tree species

**Figure 6:**
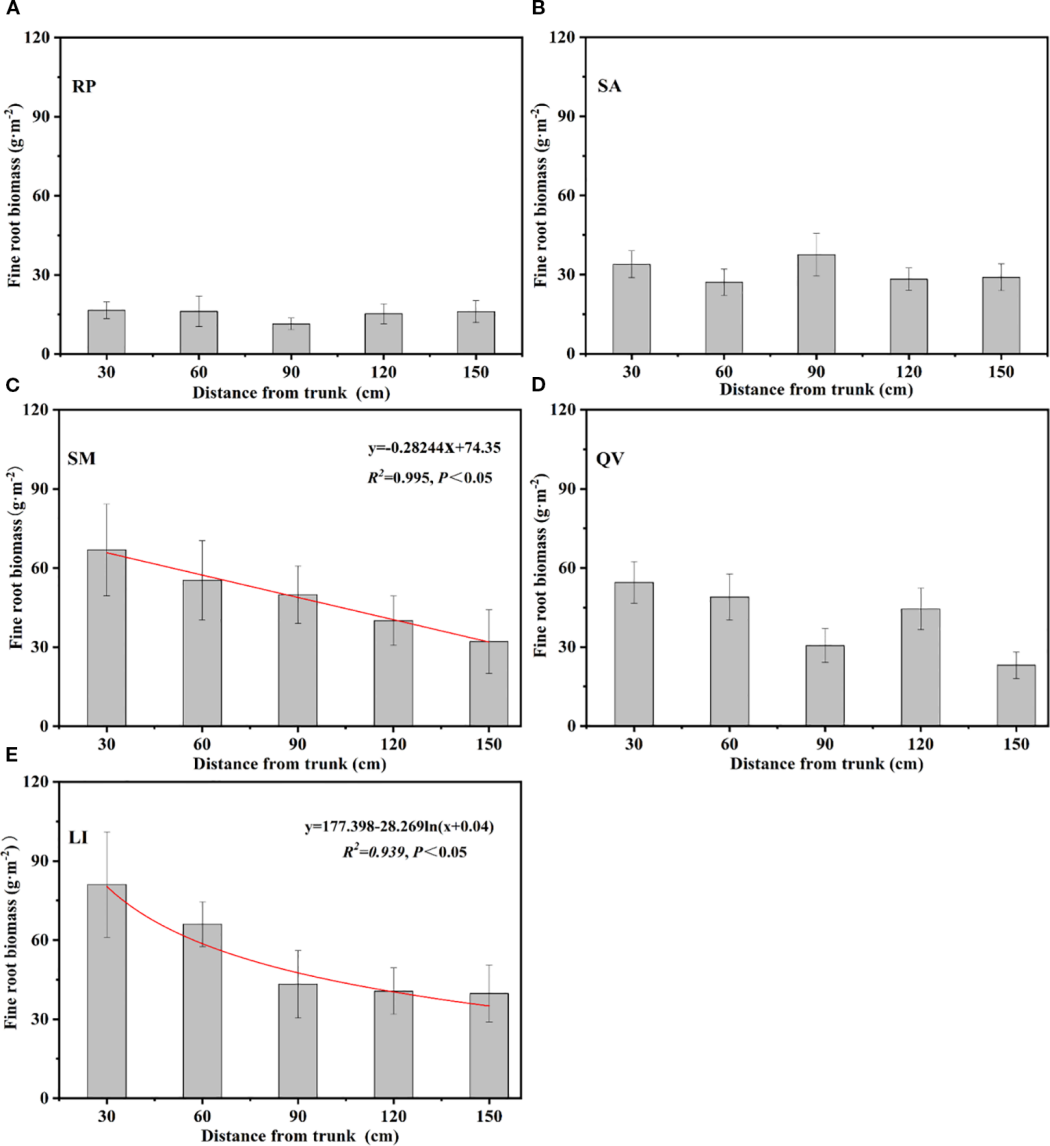
Overall variation of fine root biomass of different tree species at different distance from the trunks. **(a)** RP, **(b)** SA, **(c)** SM, **(d)** QV, **(e)** LI. RP, *Robinia pseudoacacia*; SA, *Sapium sebiferum*; SM, *Salix matsudana*; QV, *Quercus virginiana*; LI, *Ligustrum lucidum.*

The horizontal distribution patterns of fine root biomass among the five tree species revealed distinct spatial characteristics: SM, QV, and LI exhibited progressively decreasing fine root biomass with increasing distance from the trunk, with SM showing a linear reduction (R²=0.98, *P*<0.05) and LI following a logarithmic decline (R²=0.939, *P*<0.05) from the trunk center to the canopy edge (**Figure 6c, e**). In contrast, SA demonstrated a unique pattern with peak biomass (37.58 g·m^-2^) at 90 cm from the trunk (**Figure 6b**), while RP generally decreased with distance but displayed an anomalous minimum (11.41 g·m^-2^) at the 90 cm position, indicating species-specific horizontal distribution strategies in coastal saline soils (**Figure 6a**).

#### 3.2.3 The temporal dynamics of fine root biomass of different tree species

**Figure 7:**
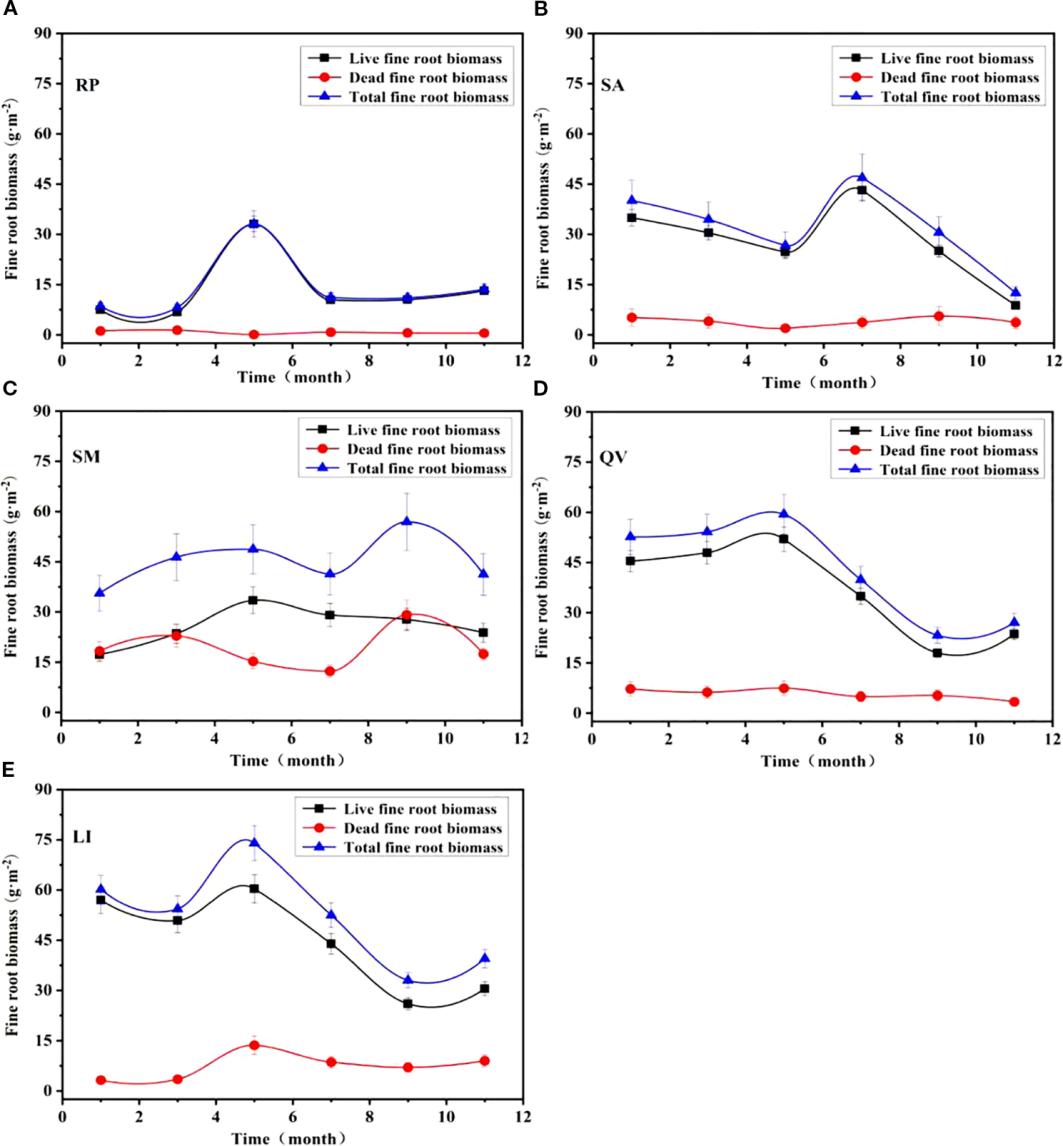
Temporal dynamics of fine root biomass in different tree species. **(a)** RP, **(b)** SA, **(c)** SM, **(d)** QV, **(e)** LI. RP, *Robinia pseudoacacia*; SA, *Sapium sebiferum*; SM, *Salix matsudana*; QV, *Quercus virginiana*; LI, *Ligustrum lucidum*.

The monthly dynamics of live fine root biomass, dead fine root biomass, and total fine root biomass exhibited distinct temporal patterns among the studied tree species, with live biomass trends mirroring total biomass variations (**Figure 7**). RP showed peak live (33.03 g·m^-2^) and total biomass (33.11 g·m^-2^) in May, while dead biomass peaked in March (1.38 g·m^-2^) (**Figure 7a**). SA demonstrated maxima for live (43.16 g·m^-2^) and total biomass (46.88 g·m^-2^) in July, with dead biomass peaking in September (5.57 g·m^-2^) (**Figure 7b**). SM showed live biomass peaking in May (33.47 g·m^-2^), while total (57.97 g·m^-2^) and dead biomass (29.19 g·m^-2^) reached maxima in September (**Figure 7c**). QV displayed synchronous peaks for live (51.96 g·m^-2^), total (59.41 g·m^-2^), and dead biomass (7.45 g·m^-2^) in May (**Figure 7d**). LI exhibited concurrent peaks for live (60.34 g·m^-2^), total (73.98 g·m^-2^), and dead biomass (8.58 g·m^-2^) in May (**Figure 7e**). These observations reveal that dead biomass peaks either coincided with live biomass maxima or occurred shortly after live biomass peaks, suggesting coordinated turnover dynamics in these coastal saline soil-adapted species.

### 3.3 The distribution characteristics of soil properties in different tree species plots

**Figure 8:**
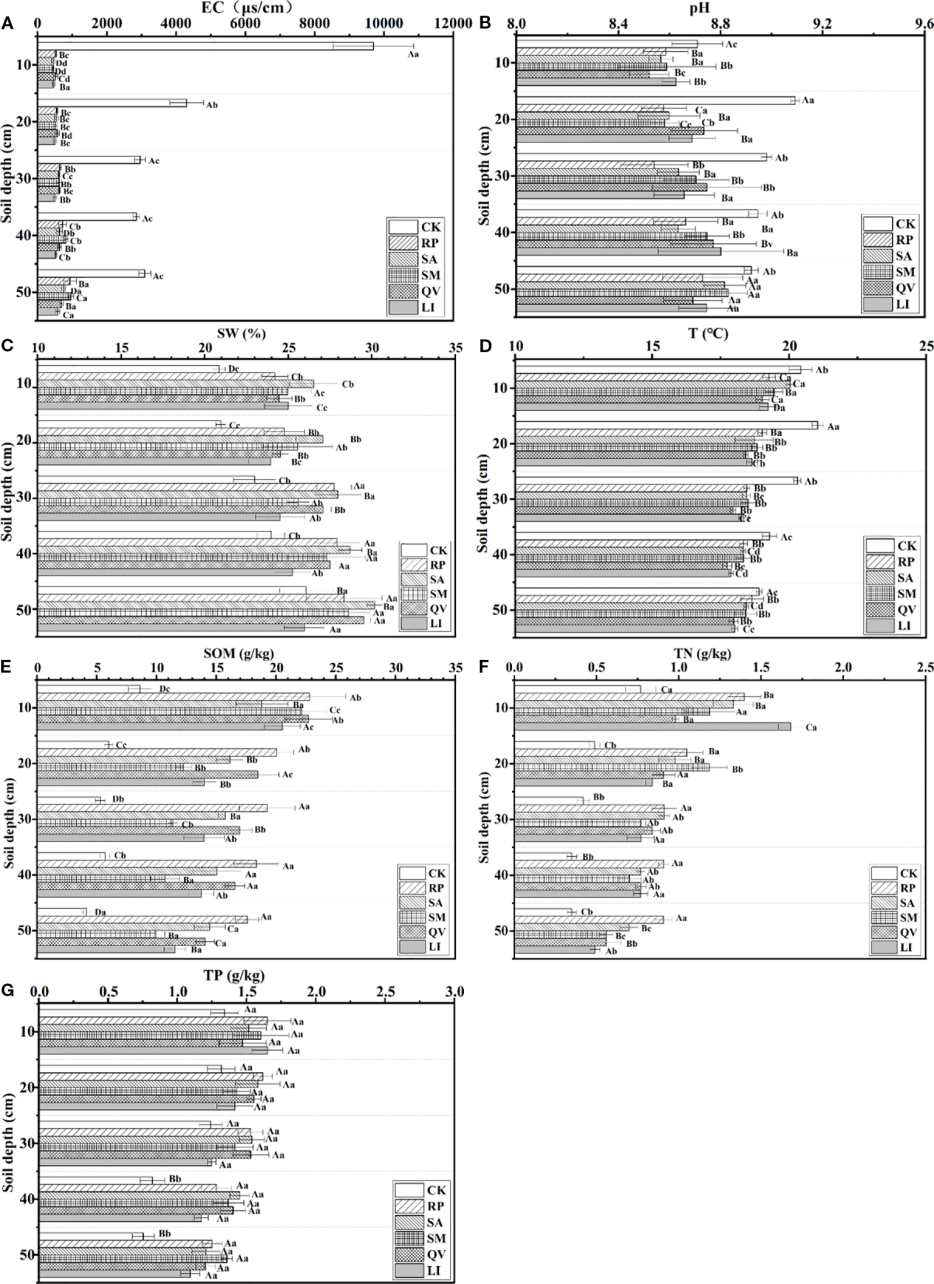
Distribution of soil properties in sample plots of different tree species. **(a)** electrical conductivity, **(b)** pH, **(c)** soil water, **(d)** temperature, **(e)** soil organic matter, **(f)** total N, **(g)** total. RP, *Robinia pseudoacacia*; SA, *Sapium sebiferum*; SM, *Salix matsudana*; QV, *Quercus virginiana*; LI, *Ligustrum lucidum;* EC, Electrical conductivity; SW, Soil water; T, Temperature: SOM, Soil organic matter: TN, Total N; TP, Total P. Different capital letters indicate the significant differences in soil physical and chemical properties between different communities at the same soil depth; and different lowercase letters indicate the significant differences in soil physical and chemical properties between different communities at the same soil depth (*P*< 0.05).

In terms of soil electrical conductivity, compared with the bare land, the soil salinity was significantly reduced after planting five tree species (*P*< 0.05, **Figure 8a**). Regarding soil pH, the pH values of the five tree species gradually increased with the increase of soil layer depth, but the pH was significantly lower compared with that of the bare land (**Figure 8b**). In terms of soil water, it content in the plots of the five tree species was significantly different from that in the bare land (*P*< 0.05, **Figure 8c**), and all of them gradually increased with the increase of soil layer depth. Concerning soil T, the T in the plots of the five tree species was lower than that in the bare land. The soil temperature in the plots of each tree species gradually decreased with the increase of soil layer depth, while the soil temperature in the bare land first increased and then decreased with the increase of soil layer depth (*P*< 0.05, **Figure 8d**). In terms of soil nutrients, the contents of soil organic matter and total nitrogen in the plots of the five tree species were both increased compared with those in the bare land, and the differences were significant (*P*< 0.05, **Figures 8e, f**). Both of them gradually decreased with the increase of soil layer depth. The total phosphorus content in the soil of each plot was higher than that in the bare land, and all of them showed a decreasing trend with the increase of soil layer depth (**Figure 8g**).

### 3.4 The relationship between fine root characteristics and environmental factors

In terms of fine root morphology, root length density and root surface area density were significantly negatively correlated with electrical conductivity and soil water (*P*< 0.05), and significantly positively correlated with soil temperature and total nitrogen (*P*< 0.05). Fine root diameter, root tissue density, specific root length and specific root surface area had a certain correlation with soil water, temperature, electrical conductivity, total nitrogen, total phosphorus and soil organic matter, but the correlations were not significant (**Table 5**). Therefore, it can be seen that electrical conductivity, soil water, temperature and total nitrogen are the main environmental factors affecting the morphological characteristics of fine roots.

**Table 5:** Correlation between the characteristics of fine roots and physical and chemical properties of soil.

Variables	SD	pH	EC	SW	T	TN	TP	SOM	LBIO	DBIO	TBIO	D	RTD	RLD	SRL	SRA	RSD
SD	1																
pH	0.735**	1															
EC	0.768**	0.572**	1														
SM	0.731**	0.377	0.711**	1													
T	-0.756**	-0.659**	-0.417*	-0.391	1												
TN	-0.817**	-0.628**	-0.551**	-0.467*	0.790**	1											
TP	-0.630**	-0.713**	-0.344	-0.187	0.557**	0.528**	1										
SOM	-0.656**	-0.628**	-0.429*	-0.339	0.562**	0.693**	0.537**	1									
LBIO	-0.519**	-0.093	-0.502*	-0.410*	0.318	0.583**	0.153	0.244	1								
DBIO	-0.213	0.019	-0.138	-0.204	0.225	0.149	-0.018	-0.225	0.365	1							
TBIO	-0.501*	-0.071	-0.462*	-0.408*	0.339	0.532**	0.121	0.127	0.950**	0.637**	1						
D	-0.325	0.063	-0.332	-0.162	0.092	0.185	0.045	0.103	0.599**	-0.091	0.465**	1					
RTD	0.017	0.204	-0.027	0.042	-0.354	-0.169	0.035	0.186	0.185	-0.249	0.070	0.455*	1				
RLD	-0.486*	-0.221	-0.453*	-0.451*	0.508**	0.579**	0.081	0.131	0.676**	0.750**	0.811**	0.016	-0.432*	1			
SRL	0.165	-0.228	0.229	0.092	-0.014	-0.060	-0.045	0.050	-0.495**	-0.110	-0.447*	-0.748**	-0.462*	-0.038	1		
SRA	0.113	-0.244	0.147	0.026	0.039	-0.022	-0.057	0.025	-0.440*	-0.084	-0.393	-0.727**	-0.556**	0.039	0.983**	1	
RSD	-0.480*	-0.194	-0.456*	-0.424*	0.489*	0.628**	0.093	0.133	0.801**	0.600**	0.864**	0.174	-0.400*	0.953**	-0.133	-0.040	1

SD, Soil depth; EC, Electrical conductivity; SW, Soil water; T, Temperature; SOM, Soil organic matter; TN, Total N; TP, Total P; LBIO, Living biomass; DBIO, Dead biomass; PRO, Productive; DEC, Decomposition rate; TUR, Turnover; D, Fine root diameter; RTD, Root tissue density; RLD, Root length density; SRL, Specific root length; SRA, Specific root surface area;RSD, Root surface-area density. *At 0.05 level (double side) significant correlation, **at 0.01 level (double side) significant correlation.

The biomass of living fine roots had an extremely significant negative correlation with soil layer depth (*P*< 0.01), a significant negative correlation with electrical conductivity and soil water (*P*< 0.05), and an extremely significant positive correlation with total nitrogen (*P*< 0.05). The biomass of dead fine roots was negatively correlated with soil layer depth, electrical conductivity, soil water, total phosphorus and soil organic matter, and positively correlated with pH, temperature and total nitrogen, but all these correlations were not significant.

The total biomass of fine roots had a significant negative correlation with electrical conductivity and soil water (*P*< 0.05), and an extremely significant positive correlation with total nitrogen (*P*< 0.01), indicating that electrical conductivity, soil water and total nitrogen are the main environmental factors affecting the biomass of fine roots.

## 4 Discussion

### 4.1 Differences in fine root morphological characteristics of different tree species

This study shows that the fine root surface area, root length density, and root surface area density of the five tree species all gradually decreased with the increase in soil layer depth (**Figure 3**). Among them, the fine root surface area, root length density, and root surface area density of LI in each soil layer were significantly higher than the corresponding three indicators of other tree species, and it showed obvious advantages in horizontal distribution. This indicates that LI has excellent resource capture ability. Its well-developed fine root system can not only efficiently absorb soil moisture and nutrients but also reduce the soil salinity in the root zone, thereby alleviating the harmful effects of salinity (Wu et al., 2024). Studies have shown that the complexity of root traits is manifested as various underground resource absorption strategies and ecological adaptability (Comas et al., 2012; Weemstra et al., 2016; Bergmann et al., 2017; McCormack and Iversen, 2019). In contrast, the values of fine root surface area, root length density, and root surface area density of *Quercus virginiana* in different soil layers and horizontal distributions are the lowest, indicating that saline-alkali soil exerts the greatest restrictive effect on its root growth. The main reasons for the above differences are as follows: After plants are planted, soil salinity tends to accumulate at the edge of the root crown, forming a “salt island” effect (Nichols et al., 2009; Chen et al., 2004). High soil salinity can reduce soil water potential, inhibit fine roots from absorbing water, thereby causing an osmotic stress effect. When plants face difficulties in water absorption, root growth is hindered. Secondly, the accumulation of a large amount of salt in plant roots can disrupt the ion balance in the cytoplasm, leading to an ion stress effect (Zhu, 2001). Therefore, high-salinity soil may restrict plant growth and alter the morphological characteristics of fine roots.

Fine root diameter and specific root length are important indicators reflecting the resource utilization strategy of plant root systems. Studies have shown that a larger fine root diameter usually means that the root system has higher mechanical strength and durability and is suitable for growing in stressful soil environments (Gong et al., 2020); a smaller fine root diameter means that the fine root turnover rate is faster and can quickly expand to adapt to the low-nutrient soil environment (McCormack et al., 2015). Meanwhile, a high specific root length, which corresponds to a larger root surface area, is characterized by strong explorative and resource-acquiring capabilities, enabling the absorption of more soil water and nutrients (Guo et al., 2016). In contrast, a low specific root length is generally associated with root thickness, stress resistance, and durability, allowing sustained growth in stable environments (Clemmensen et al., 2013). In this study, among the five tree species, Robinia pseudoacacia was found to have smaller fine root diameters and larger specific root lengths, indicating that its root system can expand rapidly. It exhibits strong adaptability and reproductive capacity in resource-poor saline-alkali soil environments, belonging to the resource-acquisitive tree species. Its root characteristics follow a “rapid” strategy for underground resource acquisition, adapting to the environment by altering fine root diameters with minimal biomass investment and high turnover rates (Eissenstat, 1992; Ostonen et al., 2007; Reich, 2014). In contrast, Quercus virginiana has larger fine root diameters and smaller specific root lengths, with relatively weaker root expansion ability and adaptability in saline-alkali soil environments, thus classified as a resource-conservative tree species. It follows a “slow” strategy for soil resource acquisition, adapting to the environment by increasing root diameters to achieve longer lifespans (Eissenstat, 1992; Ryser and Eek, 2000). In addition, besides efficient soil exploration, plant root systems must maintain a high metabolic rate to ensure the rapid acquisition of resources for survival (Bloom et al., 1985; Reich, 2014). Regardless of whether plants adopt “rapid” or “slow” resource acquisition strategies, they all follow the “conservation” principle, balancing between rapid and slow investment returns (Bergmann et al., 2020). The different strategies of root systems of different tree species for soil resource utilization, in addition to being affected by the soil environment, may be determined by the biological characteristics of different tree species themselves.

### 4.2 Differences in fine root biomass of different tree species

Fine root biomass is jointly affected by various environmental factors such as tree species type, tree age, and soil nutrients, soil water, temperature, electrical conductivity, and pH (Yuan and Chen, 2010; Liu et al., 2014). Fine root biomass can not only reflect the development degree of plant root systems but also reveal their resource acquisition strategies, ecological adaptability, and growth potential (Zhu et al., 2016). This study found that there were significant differences in fine root biomass among different tree species. Among them, the total fine root biomass of LI was the largest, reaching 273.42 g·m^-^², showing extremely strong resource acquisition ability and high resource utilization efficiency. In contrast, the total fine root biomass of RP was the smallest, only 77.05 g·m^-^², indicating its weak adaptability to the saline-alkali soil environment. In addition, the magnitude of fine root biomass can, to a certain extent, reflect the adaptability of plants to the saline soil environment (Imada et al., 2015). In this study, the fine root biomass of LI was significantly higher than that of other tree species, indicating that its root system can expand widely and quickly obtain soil moisture and nutrients in the saline soil environment, thus showing strong ecological adaptability.

The root extinction coefficient (*β*) reflects the distribution of roots in the soil; a larger root attenuation coefficient indicates a deeper root distribution (Gale and Grigal, 1987). Additionally, the core of root economics lies in the carbon input required for fine root construction and biomass accumulation, through which fine roots can explore the soil to acquire soil resources and adapt to the environment (Bergmann et al., 2020). Based on the above theories, it was found in this study that the root attenuation coefficients of QV and RP are relatively large, indicating that these two tree species are deep-rooted. Their root systems can effectively improve soil structure and enhance the absorption of water and nutrients in the soil through rapid expansion (Zhou et al., 2023). In contrast, the root extinction coefficients of LI, SM, and SA decreased successively, indicating that the root growth depths of the above three tree species are relatively shallow, and their ability to adapt to the deep soil environment is weak. In terms of horizontal distribution, the distribution patterns of fine root biomass of the five tree species were significantly different (**Figure 5**, **6**). Among them, RP and SA showed obvious pioneering characteristics by expanding the foraging range of their root systems to obtain soil resources in a larger horizontal space (Ostonen et al., 2011). On the contrary, the distribution ranges of root biomass of LI, SM, and QV were relatively small, indicating that these tree species prefer to efficiently utilize soil resources within a limited soil range rather than relying on large-scale expansion to disperse risks, reflecting the conservative characteristics of the root systems.

There were significant differences in the vertical distribution of fine root biomass among different tree species, but the general rule was that the fine root biomass gradually decreased with the increase in soil layer depth, which is consistent with the research results abroad. This study found that the fine root biomass of the five tree species was mainly concentrated in the soil layer of 0–20 cm. Among them, the proportions of fine root biomass of RP, SA, SM, QV, and LI in the total biomass were 49.05%, 57.18%, 55.18%, 40.57%, and 62.93%, respectively, and these research results were basically consistent with the research conclusions of other scholars. For example, Spanner et al. (2022) pointed out that 89% of the fine root biomass in the central Amazon forest was concentrated in the soil layer of 0–20 cm; Schwieger et al. (2021) found that the proportion of fine root biomass B in the shallow soil (0–20 cm) of forest peatlands in northeastern Germany was the highest. Similarly, the study of Liu et al. (2022) showed that about 43% of the total amount in the artificial shelter forests in Northwest China was concentrated in the soil layer of 0–40 cm. Gessler et al. (2022) also pointed out that the fine root biomass of the beech forest was concentrated in this range. This study further showed that the fine root biomass decreased exponentially with the increase in soil layer depth (except for QV). This phenomenon may be driven by the following multiple factors: First, it is determined by the vertical distribution of soil nutrients: The surface soil is usually rich in moisture and nutrients, and the resources in the deep soil gradually decrease, resulting in a significant decrease in fine root biomass with increasing depth (Batjes, 1996). Second, the advantageous conditions of the surface soil: The surface soil is relatively soft, making it easier for fine roots to grow and absorb resources (Tracy et al., 2011). Third, oxygen limitation: Root systems require aerobic respiration, and the oxygen content in the deep soil is lower, limiting the deep distribution of the root system (Chen et al., 2016). Fourth, energy cost and soil physical limitations: The growth of fine roots requires the consumption of plant energy. The compactness and hardness of the deep soil increase the difficulty of root system expansion and make the growth cost higher (Gaudinski et al., 2010). Fifth, the salt accumulation effect: In coastal saline soil, there may be a phenomenon of higher salt concentration in the deep soil, which has an inhibitory effect on plant root systems (Cebrián et al., 2023). In addition, the vertical distribution pattern of fine root biomass is also closely related to the biological characteristics of tree species themselves, and there are differences in the response ability of different tree species to the soil environment.

### 4.3 Differences in ecological adaptability of different tree species to saline soils

There are significant differences in the growth patterns of root systems among different tree species, and their distribution characteristics in the soil reflect the ecological adaptability of different tree species to the soil environment. In this study, among the fine root distribution patterns of the five tree species, the root systems of RP, SA, and SM mainly showed a horizontal distribution pattern, expanding their foraging range through horizontal spatial expansion (Li et al., 2025). This strategy allows plants to widely absorb resources from adjacent areas, reducing the limitations of scarce soil resources on growth, and demonstrating the ability to efficiently utilize shallow soil resources. Among them, SA has the widest horizontal distribution range, showing its significant advantage in the utilization of shallow resources. In contrast, the root systems of RP and QV showed a vertical distribution pattern, which has strong penetrating ability and can extend to the deep soil to utilize the niche resources at different soil depths (Geng et al., 2022). Regardless of the horizontal or vertical distribution patterns of root systems, they all conform to the “root economics space” theory, which can support the ecological adaptation of plant root systems to different soil environments.

The distribution characteristics of tree root systems are largely determined by the genetic characteristics of tree species. Previous studies have shown that each species has a “growth strategy” influenced by genetic factors and exhibits unique external morphological characteristics at specific growth stages. Therefore, by studying the root morphological characteristics and growth strategies of different tree species, we can deeply reveal the ecological adaptability of the fine root morphology of different tree species in stressful environments, providing a scientific basis for screening excellent tree species suitable for growing in special environments. However, the distribution pattern of plant root systems is not entirely determined by genetic characteristics. The soil environment also plays a crucial role in the depth and breadth of root system distribution. Even for the same plant species, its root system distribution characteristics may vary significantly under different site conditions (Zhou et al., 2024). Coastal saline soil is mainly characterized by high salinity and nutrient deficiency, and these two factors have a particularly significant impact on the distribution pattern of plant root systems (Akhter et al., 2004). In this study, the five tree species showed different root system distribution characteristics and growth strategies in the saline soil environment, reflecting their respective adaptability to the coastal saline soil environment. Based on the distribution patterns of fine root morphological characteristics and biomass of different tree species, the order of tree species suitable for saline-alkali land is as follows: LI > SM > SA > RP > QV. Due to the different root characteristics of various tree species, when planting plants in saline-alkali land, salt-tolerant native tree species with large root biomass, large growth, and high root length density should be selected. Meanwhile, arbor-shrub mixed forests composed of deep-rooted and shallow-rooted plants with well-developed taproots and lateral roots should be used to construct a three-dimensional underground root space, enabling roots to occupy the soil niche to the maximum extent and realizing the synergistic restoration of saline-alkali land by roots of different plants. Since herbaceous plants have the characteristics of fast growth and strong adaptability, they can quickly cover the ground and reduce soil water evaporation. Wild native salt-tolerant herbaceous plants such as *Salicornia europaea*, *Sesbania cannabina*, and *Suaeda salsa* are suitable for saline-alkali land. Therefore, in future greening construction and ecological restoration practices, a near-natural planting mode combining arbors, shrubs, and herbs should be fully adopted to improve green coverage and achieve ecological restoration of coastal saline land.

## 5 Conclusions

Through an in-depth study of the fine root morphological characteristics and biomass distribution characteristics of five tree species(RP, SA, SM, QV, LI), the following conclusions were drawn: There were significant differences (*P*< 0.05) in the fine root morphological characteristics (specific root length, specific root surface area, root length density, and root surface area density) of the five tree species. Among them, RP had the largest specific root length (135.38 m·g^-1^) and specific root surface area (1141.07 cm²·g^-1^), while QV had the smallest specific root length (39.17 m·g^-1^) and specific root surface area (315.22 cm²·g^-1^). The root length density and root surface area density gradually decreased with the increase in soil layer depth. Among them, LI had the largest root length density and root surface area density (18533.45 m·m^-3^, 22.08 m²·m^-3^), RP had the smallest root length density (6413.50 m·m^-3^), and QV had the smallest root surface area density (6.16 m²·m^-3^). There were extremely significant differences (*P*< 0.01) in the fine root biomass of the five tree species. Among them, the fine root biomass of LI was the largest (273.42 g·m^-2^), and that of RP was the smallest (77.05 g·m^-2^). The fine root biomass gradually decreased with the increase in soil layer depth in the vertical direction, and in the horizontal direction, the farther away from the tree trunk, the smaller fine root biomass. The root extinction coefficient indicated that QV and RP were deep-rooted tree species, while LI, SM, and SA were shallow-rooted tree species. Due to the different root survival strategies of different tree species, with the change of seasons, the peaks of living fine root biomass mainly occurred in May (RP, LI, QV), July (SA), and September (SM), and the peaks of dead fine root biomass mainly occurred in March (RP), May (LI, QV), and September (SA, SM). The peaks of living and dead fine root biomass of RP, LI, QV, and SA all showed a unimodal pattern, the peak of living fine root biomass of SM showed a unimodal pattern, and the peak of dead fine root biomass showed a bimodal pattern. Correlation analysis showed that electrical conductivity, soil water, and total nitrogen were the main environmental factors affecting the morphological characteristics and biomass of fine roots.

## Data Availability

The original contributions presented in the study are included in the article/supplementary material. Further inquiries can be directed to the corresponding author.
